# Evidence-based strategy for prevention of hidden hunger among adolescents in a suburb of Nigeria

**DOI:** 10.1186/s12889-020-09729-8

**Published:** 2020-11-10

**Authors:** Vivienne N. Ibeanu, Chinonye G. Edeh, Peace N. Ani

**Affiliations:** Department of Nutrition and Dietetics, University of Nigeria, P. O. Box 3042, Nsukka, Nigeria

**Keywords:** Adolescents, Nutrition education aids, Hidden hunger, Micronutrients, Evidence-based

## Abstract

**Background:**

Hidden hunger (micronutrient deficiencies) among adolescents are linked to impaired physical growth, poor cognitive function, low resistance to infection as well as degenerative and chronic diseases at later age. To prevent these deleterious impacts of hidden hunger, effective intervention strategy that improves nutrition knowledge and promotes healthy food choices among this age-group becomes imperative. The intervention was to evaluate the impact of a 14-page locally developed nutrition education aids on the teenagers’ knowledge of the functions, food sources and deficiencies of some micronutrients and their food choices.

**Methods:**

A one group pre-and post-intervention quasi-experimental study design was conducted with 869 adolescents (13–17 years) selected using multi-stage sampling technique in public secondary schools in a suburb of Nigeria. Using the instructional material development guidelines, the nutrition education aids were developed with nutrition facts, pictures of staple micronutrients-rich foods, and computer graphics. Baseline (pre-intervention) knowledge of nutrition and practice of food choices in relation to micronutrients were determined before exposing the students to the developed nutrition education aids and reassessing (post-intervention) them after 6 months. Data obtained were subjected to paired samples t-test using SPSS version 21.

**Results:**

The adolescents were mostly females (58.92%) aged 16–17 years (53.62%). There was significantly (*p* < 0.05) higher mean knowledge scores of general nutrition (65.77 vs. 39.61%) and food sources of nutrients (82.26 vs. 66.87%) at post-intervention compared to pre-intervention. Also, the mean knowledge of functions and deficiencies of vitamin C, folic acid, iron, calcium, and zinc were significantly (*p* < 0.05) higher at post-intervention than at pre-intervention. The study further revealed percentage increase in the proportion of respondents who consumed meat (27.72%), mango (128.20%), watermelon (152.29%), carrot (336.34%) and leafy vegetables (85.56%) daily after the intervention. In addition, the percentage of students who rarely consumed all the studied micronutrient-rich foods decreased after the intervention.

**Conclusion:**

The intervention strategy increased the nutrition knowledge and the consumption of some micronutrients-rich food sources among the adolescents. The developed nutrition education aids are recommended for use in the fight to reduce/eradicate hidden hunger among adolescents in Nigeria.

**Supplementary Information:**

The online version contains supplementary material available at 10.1186/s12889-020-09729-8.

## Background

Understanding the nature, magnitude and range of problems and solutions of micronutrient deficiency has come a long way. Micronutrient deficiency is spectra of undernutrition that occurs when intake or absorption of vitamins and minerals is too low to sustain good health and development as well as normal physical and cognitive functions. Because they develop gradually over a long period micronutrient deficiencies are referred to as *hidden hunger.* The impact of this deficiency is often not noticed until irreversible damages have already occurred in the body. These damages include but not limited to diseases like osteoporosis, osteomalacia, thyroid deficiency, colorectal cancer, and cardiovascular diseases [1], anaemia, zinc and vitamin A deficiencies. Hidden hunger makes up 7% of the global disease burden. Iron deficiency related anaemia, zinc and vitamin A deficiencies were among the 15 leading causes of disease burden and the cost associated with the care of those affected is estimated at $180 billion [2]. In addition, hidden hunger compromises socio-economic development, learning ability and productivity of an individual and of a people in general [3].

Adolescents are vulnerable group for reasons such as high nutritional requirement for growth [4] reproductive maturation, and cognitive transformations [1], their food consumption pattern and lifestyle, their risk-taking behaviours and their susceptibility to environmental influences [5, 6]. At the same time, most adolescents have inadequate knowledge about their own health, development, and nutritional needs [3]. Studies have shown that diet quality declines as children move from middle childhood into adolescence. For instance, the consumption of fruits, vegetables and milk which are the major sources of micronutrients have been shown to decrease as consumption of soft drinks increases among the adolescents. According to the World Health Organization [3] adolescents do not satisfy their daily requirements of these micronutrients of importance: iron, calcium, vitamins A and C and folate, probably because of high rates of smoking, alcohol consumption and use of illicit drugs observed amongst them. Smokers for example have been reported to consume less fruits and vegetables [7]. Also, many adolescents are ignorant of some healthy and non-healthy foods [8] and poor knowledge of nutrition is among the multi-sectoral factors involved in development of malnutrition [9]. Nonetheless, teenagers are not traditionally considered as nutritionally at-risk group in many communities.

In Nigeria, the prevalence of micronutrient deficiencies remains a public health concern especially among the adolescents. Previous studies in Enugu state reported consistently high prevalence of some micronutrient deficiencies among adolescents. In 2003, a study of 600 adolescents showed that 40% males and 32% females had low plasma concentrations of vitamin A (< 20 microg/dL) and 47% had low plasma concentrations of vitamin C [10]. Another study by Ayogu et al. [11] reported 64% anaemia, 44% vitamin A deficiency (VAD), and 40% anaemia and VAD among 400 adolescents in Enugu state. Onoja et al. [12] surveyed a sample of 647 adolescents and reported that 40% were both anemic and vitamin A deficient whereas 57% had multiple malnutrition. There is opacity of information on zinc, folate, and calcium deficiencies among adolescents in Enugu state. Despite the availability and accessibility of variety of fruits, and vegetables in the state; these researchers to a large extent attributed the high prevalence of micronutrient deficiencies among the adolescents to ignorance of rich-food sources of micronutrients resulting in poor food choices and consumption pattern. Irrespective of the high prevalence of micronutrient deficiency among the adolescents in Enugu state, limited studies have been carried out to address this public health issue. Nutrition intervention programmes in Enugu State focus mostly on maternal and child health. In line with the above, it becomes imperative to provide evidence-based strategy for prevention of hidden hunger among adolescents in the state.

Nutrition education is a vital tool in school because the knowledge received would empower the adolescents to positively influence their parents’ food choices and practices. Food-base nutrition education approach is sustainable and could be a simple method of preventing and treating micronutrient deficiencies. Previous studies demonstrated that nutrition education programmes can improve nutrition knowledge and food consumption pattern of a population [13–16]. Nutrition education is any combination of educational strategies designed to facilitate voluntary adoption of food choices and other food and nutrition-related behaviours conducive for good health and well-being [17]. It aims at stimulating behavioural change towards improve dietary diversity and diet quality [18]. Good behavioural change communication strategies are required to elicit permanent changes, and this could be achieved by using appropriate communication aids in schools. The present study was carried out to assess the use of locally developed nutrition education aids as a strategy to address hidden hunger among adolescents in Enugu state. The nutrition education aids developed were in accordance with the Food and Agricultural Organization/World Health Organization [19] guidelines on the use of bright colours and photographs to make the materials easier to read, understand and more appealing to the audience.

## Methods

### Area and design of study

Quasi-experimental study design with one intervention group was used to study randomly selected adolescents from public secondary schools in Nsukka local government area (LGA) of Enugu State, Nigeria. Nsukka is in south-eastern part of the country and a suburban area on hilly savannah vegetation. Nsukka has an area of 1810km^2^ and a population of 309,633 [20]. The inhabitants are mostly civil servants and traders. There are 30 public secondary schools in Nsukka LGA; 21 of which are co-educational, 5 all girls’ and 4 all boys’ schools.

### Study population, sample size and sampling technique

The study population was all the senior secondary school students (adolescents) in the 30 public secondary schools in Nsukka LGA. The adolescents targeted for this intervention were between 13 and 17 years of age. The sample size was determined using the formula for one-group before and after study (paired t-test) proposed by Rosner [21].

$$ n=\frac{{\left(\mathrm{Z}\upalpha /2+\mathrm{Z}\upbeta \right)}^2}{{\left(\frac{E}{\mathrm{S}\left(\Delta \right)}\right)}^2} $$

Where

- n = Minimum sample size.
- Z_α/2_ = Critical value of the normal distribution at α/2 (for 95% confidence interval, the critical value is 1.96)
- Z_β_ = Critical value of the normal distribution at β (for a power of 80%, the critical value is 0.84).
- E = Effect size (0.1).
- S(Δ) = Standard deviation of change in the outcome, (1.0).

Using the formula and conditions above, the minimum sample size required to detect a difference between two means was calculated as 785. This was increased by 84 (≈ 10%) to adjust for uncertainties such as attrition. Therefore, the final calculated sample size for this study was 869.

**Fig. 1:**
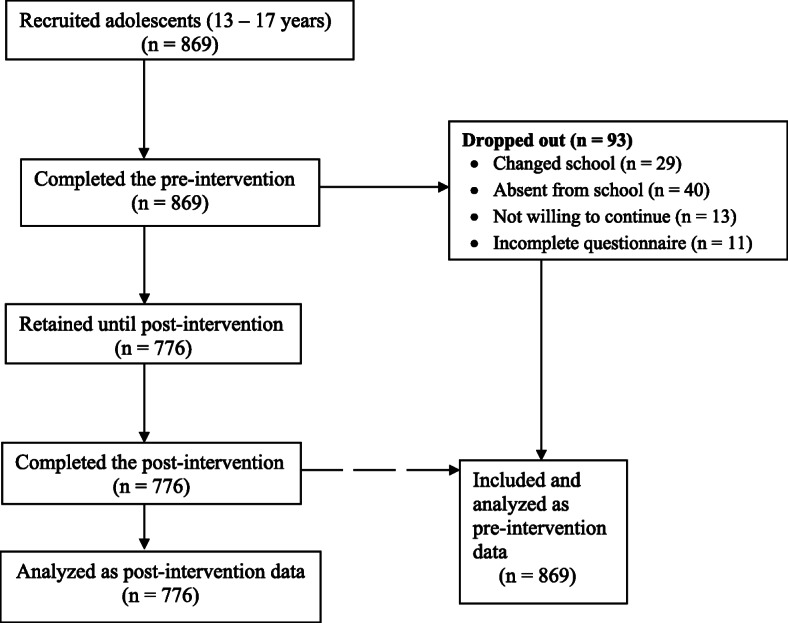
Flow chart of the study design

A multi-stage random sampling technique was adopted. First, Nsukka LGA was stratified into towns. The names of public schools in each town were compiled from the local government educational zone. In the second stage, 9 (5 co-educational, 2 girls’ and 2 boys’ schools) out of the 30 public schools were randomly selected. The 9 schools were in different towns within the LGA. This helped to curtail the effect of contamination on the respondents. There was minimized possibility of students earlier exposed to the nutrition education aids passing the information to others whose pre-intervention data were yet to be collected. In the last stage, systematic sampling was used to select every third student (within 13–17 years) in each class until the required number was reached. A minimum of 95 students were selected from each school giving a total of 869 students. The participants were students in senior secondary 1 and 2. The senior secondary 3 (SS3) students were excluded from the study because they were preparing for O’level (West African Examination Council) examination. In addition, this group of students stops attending school once the examination ends. Out of the 869 students who participated in the pre-test, 776 adolescents took part in the post-test because some dropped due to reasons such as change of school, absenteeism, and unwillingness to continue (Fig. 1).

### Instrument for data collection

Data were collected using self-administered structured and validated questionnaire. The questionnaire was validated by experts in the nutrition education in the Department of Nutrition and Dietetics, University of Nigeria, Nsukka. A pre-test was carried out in a pilot study consisting of 87 adolescents in 2 randomly selected schools in Nsukka LGA. The schools were different from the ones used for this study. The outcome of the pilot study gave room for necessary modifications made to the questionnaire before the commencement of the study. The questionnaire had three parts. Part A assessed the socio-demographic characteristics and lifestyle of the respondents. Part B was divided into three sections which evaluated nutrition knowledge. Part B (1) comprised 11 nutrition facts which tested the students’ knowledge on general nutrition and hidden hunger. Part B (2) had 34 nutrition facts to assess their knowledge of the main food nutrients in some commonly consumed foods. While Part B (3) had 5–7 nutrition facts on the main functions, signs, and symptoms of deficiencies of vitamins A and C, folic acid, iron, calcium, and zinc. Each correct answer on the knowledge scale had one point. The performance of each respondent at pre-and post-intervention was graded and the final score converted to percentage by dividing with the number of nutrition fact tested and multiplying by 100. The percentage scores were categorized as ***very good knowledge*** (100–70%), ***good knowledge*** (69–40%) and ***poor knowledge*** (< 40%). The Part C of the questionnaire investigated the consumption pattern of micronutrient-rich foods (egg, milk, liver, fish, meat, nut, paw-paw, orange, mango, carrot, watermelon, guava, pineapple and leafy vegetables) among the adolescents. Questions like *how often do you consume the following foods*? were asked and options given included *daily*, *2 to 3 times/week*, *once a week*, and *rarely.* Additional file 1 shows this in more detail. Eight hundred and sixty-nine (869) students participated in the pre-intervention phase of the study which involved filling of questionnaire. It took each student between 20 and 25 min to fill the questionnaire. Cronbach’s alpha test was applied to test the reliability of the questionnaire over time and the mean value (0.85) obtained was within the acceptable range of 0.65 and 0.90 [22].

### Outcome measures

The main outcomes were increase in the nutrition knowledge scores in relation to micronutrients (vitamins A and C, folic acid, iron, calcium, and zinc) and consumption of staple micronutrient-rich foods.

### The nutrition intervention

The intervention strategy used in this study focused on nutrition education targeting the adolescents. The nutrition education was conducted in the 9 selected schools using the developed nutrition aids with the aim of: (i) improving dietary diversity and food choices, (ii) increasing intake of foods rich in specific micronutrients and (iii) decreasing intake of inhibitors of micronutrient absorption. The nutrition education aids developed with nutrition facts, pictures of micronutrients-rich foods and computer graphics according to instructional material development guidelines [19] were compiled into a 14-page booklet. The intervention was delivered through in-class experimental lessons. The components of the intervention were as follows:

(i) definition of food nutrients, classes and amount required,
(ii) food sources of micronutrients of interest in this study,
(iii) functions of the micronutrients,
(iv) signs and symptoms of the micronutrient deficiencies,
(v) inhibitors of the micronutrient absorption.

In each school, the adolescents were given experimental lessons 2 days/week for 3 weeks using the developed nutrition education aids. Each lesson lasted for 40 min. Six months after the intervention, the researchers went back to the schools to reassess the respondents (post-intervention) using the same questionnaire and mode of administration but the sample size was less (776). This was because some of the respondents changed school or were absent or unwilling to participate. The study lasted from September 2016 to July 2017.

### Statistical analysis

Data were analyzed using the Statistical Package for Social Sciences (SPSS), version 21. Statistical significance was set at an alpha level of < 0.05. The effect of the developed nutrition education aids was measured with paired samples t-test. First, mean of pre-and post-intervention scores of nutrition knowledge questions were calculated. These data were subjected to paired samples t-test to evaluate the difference between the mean scores at pre- and post-intervention. The scores were further categorized and summarized in frequencies and percentages. Respondents’ consumption pattern of micronutrient-rich foods at pre- and post-intervention was summarized as percentage change.

## Results

Eight hundred and sixty-nine (869) adolescents (13–17 years) randomly selected from public secondary schools participated in the pre-intervention whereas 776 participated in the post-intervention. Background information and food consumption pattern of the respondents were shown in Table 1. A total of 41% of the adolescents were males, more than half (53.62%) were between 16 and 17 years old. Most parents (53.16%) earned monthly income ranging from 31,000 to 50,000 Naira (69–111 US Dollar).

The prevalence of alcohol consumption among the respondents was 13.69%. Regarding meal skipping habit, majority (64.79%) skipped meal and 23.27% reported skipping meal every day. Breakfast (73.18%) was the most skipped meal of the day and weight reduction (56.48%) was the major reason for skipping meal.

**Table 1:** Background information and food consumption pattern of the respondents

Variables	Frequency	Percentage (%)
**Sex**		
Male	357	41.08
Female	512	58.92
**Total**	**869**	**100.00**
**Age-group**		
13–15 years	403	46.38
16–17 years	466	53.62
**Total**	**869**	**100.00**
**Parents’ monthly income**		
< 18,000	3	0.35
18,000–30,000	22	2.53
31,000–50,000	462	53.16
51,000–100,000	304	34.98
> 100,000	78	8.98
**Total**	**869**	**100.00**
**Alcohol consumption**		
Yes	119	13.69
No	750	86.31
**Total**	**869**	**100.00**
**Meal skipping habit**		
**Skip meals**		
Yes	563	64.79
No	306	35.21
**Total**	**869**	**100.00**
**Frequency of meal skipping**		
Everyday	131	23.27
< 2 times a week	78	13.85
2–3 times a week	206	36.59
> 3 times a week	148	26.29
**Total**	**563**	**100.00**
**Meal mostly skipped**		
Breakfast	412	73.18
Lunch	23	4.09
Dinner	128	22.73
**Total**	**563**	**100.00**
**Reasons for skipping meal**		
No food	36	6.40
To reduce weight	318	56.48
Too early to eat	103	18.29
Late for classes	28	4.98
No appetite	78	13.85
**Total**	**563**	**100.00**

Categorized scores and mean differences of the knowledge of general nutrition and micronutrients at pre-and post-intervention are shown in Table 2. The proportion of respondents who had very good knowledge of general nutrition and main nutrients in some commonly consumed foods increased from 32.34 and 42.35% at pre-intervention to 69.33 and 65.85% at post-intervention, respectively. The percentage of those who had very good knowledge of functions and deficiencies of the micronutrients studied also increased from 7.25–20.48% at pre-intervention to 24.23–54.64% at post-intervention. Likewise, the respondents who had poor knowledge of the nutrition facts tested decreased after the intervention. There was significantly (*p* < 0.05) higher mean knowledge scores of general nutrition, food sources, functions and deficiencies of vitamin C, folic acid, iron, calcium and zinc at post-intervention compared to pre-intervention. Also, significantly (*p* < 0.05) higher mean knowledge scores were observed at post-intervention compared with pre-intervention for all the nutrition facts tested except vitamin A which had high score but the difference was not significant (*p* > 0.05).

**Table 2:** Categorized scores and mean difference of knowledge of general nutrition and micronutrients at pre-and post-intervention

Intervention	No of Nutrition facts tested	Very good knowledge n (%)	Good knowledge n (%)	Poor knowledge n (%)	Mean ± SD	^**a**^Mean difference	*P*-value
**General nutrition**							
Pre-intervention	11	281 (32.34)	422 (48.56)	166 (19.10)	39.61 ± 13.32	−26.16	< 0.001***
Post-intervention		538 (69.33)	196 (25.26)	42 (5.41)	65.77 ± 14.01		
**Main nutrients in common foods**							
Pre-intervention	34	368 (42.35)	309 (35.56)	192 (22.09)	66.87 ± 18.97	−15.39	< 0.001***
Post-intervention		511 (65.85)	187 (24.10)	78 (10.05)	82.26 ± 11.45		
**Vitamin A: functions and deficiencies**							
Pre-intervention	7	178 (20.48)	184 (21.18)	507 (58.34)	32.84 ± 15.81	−16.64	0.134
Post-intervention		361 (46.52)	296 (38.14)	119 (15.34)	49.48 ± 15.78		
**Vitamin C: functions and deficiencies**							
Pre-intervention	5	102 (11.74)	211 (24.28)	556 (63.98)	23.59 ± 15.98	−25.22	0.039*
Post-intervention		424 (54.64)	206 (26.55)	146 (18.81)	48.81 ± 16.36		
**Folic acid: functions and deficiencies**							
Pre-intervention	5	90 (10.36)	114 (13.12)	665 (76.52)	6.90 ± 4.88	−33.51	< 0.001***
Post-intervention		246 (31.70)	358 (46.13)	172 (22.17)	40.41 ± 9.96		
**Iron**: **functions and deficiencies**							
Pre-intervention	5	100 (11.51)	128 (14.73)	641 (73.76)	28.12 ± 14.75	−23.93	0.035*
Post-intervention		306 (39.43)	214 (27.58)	256 (32.99)	52.05 ± 14.01		
**Calcium**: **functions and deficiencies**							
Pre-intervention	6	112 (12.89)	209 (24.05)	548 (70.62)	17.56 ± 13.15	−24.34	0.049*
Post-intervention		291 (37.50)	322 (41.49)	163 (21.01)	41.90 ± 18.93		
**Zinc**: **functions and deficiencies**							
Pre-intervention	5	63 (7.25)	162 (18.64)	644 (74.11)	7.71 ± 9.30	−18.76	0.048*
Post-intervention		188 (24.23)	314 (40.46)	274 (35.31)	26.46 ± 15.37		

*p < 0.05, *** *p* < 0.001

^a^Mean difference = Pre-intervention – Post-intervention

**Fig. 2:**
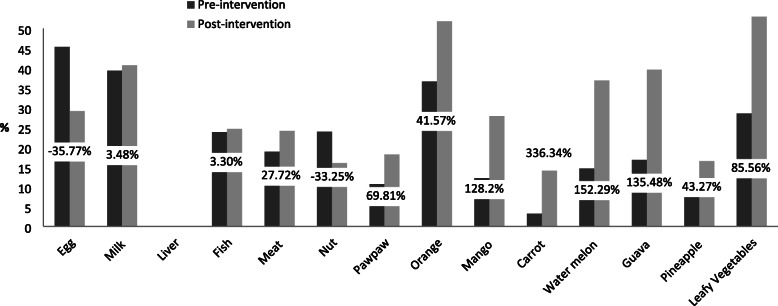
Percentage change in frequency of consumption (daily) of micronutrient-rich foods

**Fig. 3:**
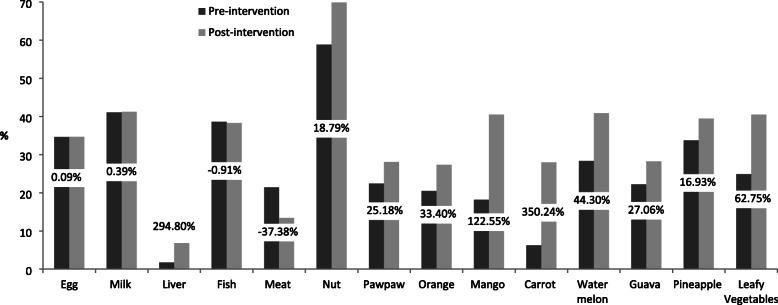
Percentage change in frequency of consumption (2–3 times weekly) of micronutrient-rich foods

**Fig. 4:**
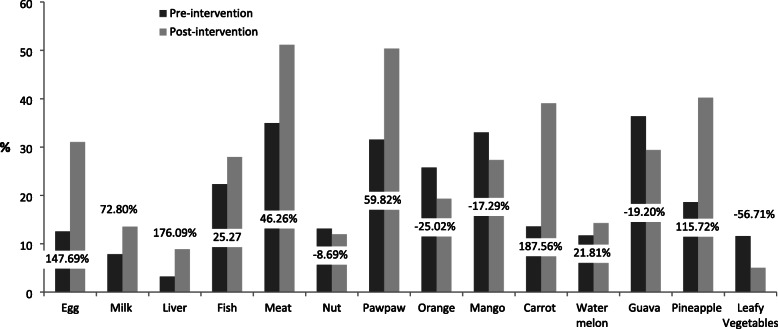
Percentage change in frequency of consumption (once a week) of micronutrient-rich foods

**Fig. 5:**
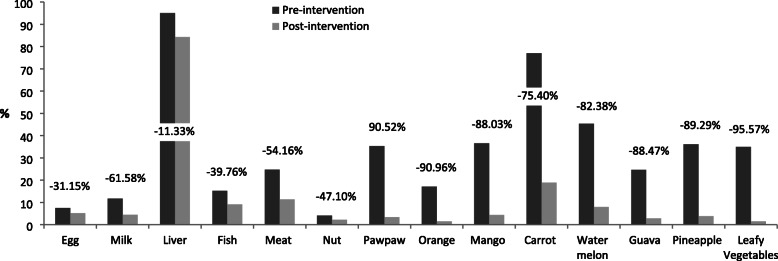
Percentage change in frequency of consumption (rarely) of micronutrient-rich foods

The percentage changes in consumption of micronutrient-rich foods among the respondents were presented in Figs. 2, 3, 4 and 5. The Fig. 2 revealed that at post-intervention, there was percentage increase in daily consumption of all the food items except for egg and nuts which decreased by − 16.22% and − 7.96%, respectively. There was high percentage increase in daily consumption of micronutrient-rich foods such as carrot (336.34%), watermelon (152.29%), guava (135.48%), mango (128.20%), pawpaw (69.81%), and leafy vegetables (85.56%) after the intervention (Fig. 2). Those who consumed liver, mango, carrot, watermelon, orange, and leafy vegetables 2 to 3 times per week increased by 294.80, 122.55, 350.24, 44.30, 44.30 and 62.75%, respectively after the intervention (Fig. 3). The proportion of respondents who consumed nuts, orange, mango, guava and leafy vegetables only once in a week decreased by (− 8.69%), (− 25.02%), (− 17.29%), (− 19.20%) and (− 56.71%), respectively (Fig. 4). Figure 5 showed that the percentage of the students who rarely consumed all the micronutrient-rich foods decreased after the intervention.

## Discussion

This one group intervention quasi-experimental study design achieved high increase in nutrition knowledge in a sample of 776 adolescents (13–17 years) in a suburban of Nigeria who found the developed nutrition education aids enjoyable and engaging. Most of the respondents had poor knowledge of functions and deficiencies of the micronutrients of interest in this study before the intervention was instituted. This observation was surprising because Food and Nutrition is part of the junior secondary school curriculum in Nigeria. The answer may lie on the assertion of Wang et al. [14] that tutors need to be well-trained and familiarized with the programmes (curriculum) aims, procedures, and tools for them to impact the required knowledge. Unfortunately, in the curriculum of the Teacher Training Colleges in the country, Food and Nutrition is not a core course. Also, inadequate use of appropriate teaching aids when lessons are delivered in secondary schools could be a contributory factor. Teaching aids are known to help capture attention and improve learning capacity especially among young people.

However, the mean nutrition knowledge scores of all parameters tested significantly increased at post-intervention compared to pre-intervention. Research on effect of nutrition education intervention on nutrition knowledge of young people were consistent in reporting increased knowledge score at post-intervention. Their recapitulations are delineated in various intervention studies [14, 16, 23–25]. The significant increases could be attributed to the gain in knowledge after the exposure to the developed nutrition education aids.

Only a few of the respondents in this study consumed fruits and vegetables daily prior to the intervention. This could be attributed to skipping of meals especially breakfast observed among more than half of the study population. According to Ani et al. [26] adolescents in Enugu state practiced poor dietary habit such as snacking on junk foods and skipping breakfast which could expose them to malnutrition. Other studies have shown that children who skipped meals had insufficient intake of fruits and vegetables compared to the ones with regular meal eating pattern [27, 28]. Low intake of these foods had been linked with different micronutrient deficiencies observed in different studies [29–31].

In this study, we found that the nutrition education intervention improved healthy food choices among the adolescents and reduced their choices of foods that inhibit micronutrient absorption. The proportion of those who consumed the targeted micronutrient-rich foods daily increased after the intervention. A comparison of the pre-and post-intervention data also revealed percentage decrease in the proportion of adolescents who rarely consumed all studied micronutrient-rich foods after the intervention. This finding is in line with earlier studies in China [14, 32], Malaysia [25] and Turkey [33] which showed that school-based prevention interventions can lead to an improvement in dietary behaviours by increasing the consumption of healthy foods and decreasing the consumption of unhealthy foods and people with adequate nutritional knowledge are most likely to consume fruits and vegetables [34].

We acknowledge the following limitations of the study. First, the study was conducted in public secondary schools hence, the findings cannot be generalized to adolescents who are in private schools due to variations that may exist in their characteristics. Secondly, data were collected between two school terms. This might have contributed to reduction in the sample size at post-intervention. Again, while the nutrition knowledge majorly influenced dietary choices of the adolescents, we acknowledge that other factors (such as inability to recall foods consumed prior to the intervention) could have contributed to the increased consumption reported among the adolescents.

## Conclusion

There was increased knowledge of micronutrient rich-foods, functions and deficiencies of vitamins A and C, folate, iron, calcium, and zinc; as well as improved healthy food choices among the adolescents after the intervention. This study provides evidence-based support for prevention of hidden hunger among adolescents using locally developed nutrition education aids. The innovative and interactive nutrition education aids developed in this study are strongly recommended for use in the fight against hidden hunger among adolescents. Our results might be interpreted in a wider scope for Nigerian adolescents residing in similar environment. Based on the results, in future, nutrition education aids addressed to other age groups can be developed by other researchers. The findings of this study could be implemented as public health action by the Nigerian government.

## Supplementary Information

**Additional file 1.** Questionnaire. Questionnaire for data collection on the development and testing of nutrition education aids for hidden hunger.

## Data Availability

The dataset used and/or analysed during the current study are available from corresponding author in reasonable request.

## Abbreviations

SPSS: Statistical Package for Social Sciences
LGA: Local government area
FAO/WHO: Food and Agricultural Organization/ World Health Organization
VAD: Vitamin A deficiency
