# Supplementary material for: Evidence-based strategy for prevention of hidden hunger among adolescents in a suburb of Nigeria
Source: BMC Public Health. 2020 Nov 10;20:1683. doi: 10.1186/s12889-020-09729-8 (PMC7654145; doi:10.1186/s12889-020-09729-8)
Supplement: Supplementary file 1 — Additional file 1. Questionnaire. Questionnaire for data collection on the development and testing of nutrition education aids for hidden hunger. [file 12889_2020_9729_MOESM1_ESM.pdf]

QUESTIONNAIRE

Development and Testing of Nutrition Education Aids for Hidden Hunger in Adolescents in Secondary Schools in Nsukka Local Government Area, Enugu State, Nigeria

PART A

Tick (√) the boxes with most appropriate answer to the questions below

PERSONAL DATA:

1. Name of your School: ____
2. Identification Number: ____
3. Sex: (a) Male ☐ (b) Female ☐
4. Age: (a)13 – 15 years ☐ (b)16 – 17 years ☐
5. Class: (a) SS1 ☐ (b) SS2 ☐
6. 6. Mother's Education

(a)First School Leaving Certificate ☐ (b) Senior Secondary School Certificate ☐ (c) National Certificate on Education/Ordinary National Diploma ☐ (d) Graduate ☐ (e) Postgraduate ☐

7. Father's Education

(a) First School Leaving Certificate ☐ (b) Senior Secondary School Certificate ☐ (c) National Certificate on Education/Ordinary National Diploma ☐ (d) Graduate ☐ (e) Postgraduate ☐

8. Mother's Occupation: (a) Farming ☐ (b) Trading ☐ Civil Servants ☐ Others please specify:_______________

9. Father's Occupation: (a) Farming ☐ (b) Trading ☐ (c) Civil Servants ☐ others please specify:_______________

10. Income of parents – (a) Below ₦18,000 ☐ (b) ₦18000 – ₦30,000 ☐ (c)₦31,000 – ₦50,0000 ☐ (d)₦ 51,000- ₦ 100,000 ☐ (e) Above ₦100,000 ☐

11. Source of Food: (a) Boarding School ☐ (b) Home with parents ☐ (c) Eateries ☐ (d) Live alone ☐ (e) Others please specify:_____________

12. If you live at home, what is the source of family food: (a) Only from market ☐ (b) Only from farm ☐ (c) Partly from market and partly from farm

13. Religion :(a) Christianity ☐ (b) Muslim ☐ (c) Traditional Worshipper ☐ (d) Others please specify_________________

14. Do you smoke: (a) Yes ☐ (b) No ☐

15. Do you take alcohol :(a) Yes ☐ (b) No ☐

16. Do you skip meal: (a) Yes ☐ (b) No ☐

17. If yes to question 16 how often: (a) Everyday ☐ (b) 2 – 3 times a week ☐ (c) More than 3 times ☐

18. Which meal of the day do you normally skip: (a) Breakfast ☐ (b) Lunch ☐ (c) Dinner ☐

19. Reasons for skipping meal: (a) No food ☐ (b) To reduce weight ☐ Too early to eat (d) Late for classes ☐ (e) Do not like food ☐(f) Do not have appetite ☐(g) Other please specify________________

20. How much are you given as pocket money in a month: (a) Below ₦ 1000☐ (b) ₦ 1000 - ₦ 5000 ☐ (c) ₦ 5100 - ₦ 10000 ☐(d) Above ₦ 10000 ☐

21. What do you mostly spend your pocket money on: (a) food ☐(b) snacks ☐(c) beverages ☐ (d) books ☐(e) clothes ☐f) Others please specify:-------------------------------

Part B (1) General Knowledge on Nutrition

1. What are food nutrients: (a) Energy giving food ☐ (b)Body protective foods ☐(c) Chemical substances found in foods ☐ (d) Foods that build the body ☐
2. Which of the following is not a food nutrient (a) Water ☐ (b)Energy ☐(c) carbohydrates ☐ (d) Protein ☐ (e) Vitamins ☐(f) Minerals ☐g) Fats and oil ☐
3. What are micro nutrients (a) Nutrients that our body require in larg☐(b) Foods that give energy☐ (c) Nutrients that our body require in small quantities ☐d) Nutrients that are not important ☐
4. Which of these is not a micronutrient (a) Vitamin ☐(b) Protein ☐(c) Mineral ☐
5. Which of these is not an example of vitamin: (a) Retinol ☐(b)Folate ☐Ascobic acid ☐) Zinc ☐
6. Which of these is not an example of mineral (a) Zinc ☐(b) Iron ☐c) Thiamin ☐) Calcium ☐
7. Which of these micronutrients is important for adolescent girls: (a) Zinc ☐ (b) Iron ☐ (c) Calcium ☐ (d) Folate ☐
8. Which of these is not a fat soluble vitamin: (a) vitamin A☐ (b) vitamin D☐ (c) vitamin C ☐ (d) vitamin E ☐
9. Which of these is not a water soluble vitamin: (a) Thiamin ☐ (b) vitamin C☐ (c) vitamin K ☐ (d) Folate ☐
10. Which of these micronutrients is not a mineral: (a) Niacin☐ (b) Calcium ☐ (c) Iron ☐ (d) Potassium ☐
11. Hidden hunger is lack of: (a) Carbohydrate ☐ (b) Micronutrient ☐(c) Macronutrient ☐ (d) Protein ☐

Part B (2) General Knowledge on Nutrition

Tick (√) the main food nutrient in each of the listed food contains

S/N	Names of food	Carbohydrate	Protein	Vitamin	Mineral	Fat/oil
1	Bambara nut pudding (okpa)					
2	Rice					
3	Beans (cowpea)					
4	Yam					
5	Cassava					
6	Semolina					
7	Orange					
8	Tomatoes					
9	Cucumber					
10	Milk					
11	Soft drinks					
12	Pasta and noodles					
13	Fish					
14	Egg					

15	Meat					
16	Water melon					
17	Pineapple					
18	Paw paw					
19	Margarine					
20	Green leafy vegetable e.g. (ugu)					
21	Ground nut					
22	Banana and plantain					
23	Cocoyam					
24	Potato					
25	Soursop					
26	Cashew					
27	Tiger nut					
28	Avacado pear					
29	Maize					
30	Pigeon pea (fiofio)					
31	Carrot					
32	Green beans					
33	Okro					
34	Garden egg					

PART B (3)

KNOWLEDGE OF FUNCTIONS AND DEFICIENCY EFFECTS OF MICRONUTRIENTS

Look carefully at the functions of micronutrients listed on your left and tick (√) the main micronutrient responsible for each function

S/N	Functions	Iron	Calcium	Zinc	Vit. A	Vit. C	Folate
1	Needed for proper development of the brain cells						
2	Prevents lack of blood (Anaemia)						
3	Lack can cause Tiredness (fatigue)						
4	Lack causes inability to concentrate						
5	Lack can result to impaired physical and cognitive development						
6	For development of healthy bone and teeth						
7	Plays a role in constriction and relaxation of blood vessels						
8	Helps nerve impulse transmission						
9	Helps muscle contraction						
10	Helps in secretion of hormones like insulin						
11	Helps to maintain the acid base balance						
12	Stops bleeding from wound or injury						
13	Essential for growth and development						
14	Involves in enzymatic reactions in the body						
15	Helps in the movement of vitamin A in the liver to maintain blood concentrations						
16	Involve in the development of foetus during pregnancy						

S/N	Functions	Iron	Calcium	Zinc	Vit. A	Vit. C	Folate
17	Important for sexual maturation						
18	Prevents anaemia in which red blood cells are abnormally large						
19	Linked to academic achievements						
20	Reduce the risk of heart diseases and stroke						
21	Prevents deformity of new born baby						
22	Maintains old cells and creates new ones						
23	Helps in the formation of collagen						
24	Protects the gums and mouth from infection						
25	Helps wounds to heal more quickly						
26	An important antioxidant						
27	Needed in iron absorption						
28	Encourages growth						
29	Needed to maintain normal vision						
30	Reduces cells that causes cancer significantly						
31	Required for normal functioning of the immune system						
32	Required for maintaining the epithelial of mucous membranes						
33	Enhances the absorption of vitamin A						

PART C
Consumption Profile

Tick (√) on how often do you consume the following foods

Food	Everyday	2 – 3 times (week)	Once a week	Rarely
(a) Energy Giving Foods				
Potatoes				
Cereals (maize, rice, etc)				
Bread				
Soft drinks				
Cassava				
Yam				
Pasta and noodles				
(b) Body Building Foods				
Fish				
Meat				
Poultry				
Egg				
Liver				
Milk				
Beans (cowpea, fiofio, akidi)				
Yogurt				
Nuts (groundnut, cashew nut)				
(c) Body Protective Foods				
Mango				
Orange				
Water melon				
Apple				
Guava				

Food	Everyday	2 – 3 times (week)	Once a week	Rarely
Pineapple				
Paw paw				
Green leafy vegetables				
Carrots				
Cabbage				
Pumpkin				
Fruits juice				
Star apple (udala)				
Soursop				
Pear (avocado, local pear)				
Cashew				
Tiger nut				
Okro				
